# Fatal Infection Caused by *Chromobacterium violaceum*: A Case Report from a Tertiary Care Hospital in Bangladesh

**DOI:** 10.1155/2019/6219295

**Published:** 2019-04-23

**Authors:** Sadia Sharmin, Aflatun Akter Jahan, S. M. Mostofa Kamal, Protim Sarker

**Affiliations:** ^1^Department of Microbiology, Anwer Khan Modern Medical College, Dhaka, Bangladesh; ^2^Intensive Care Unit, Anwer Khan Modern Medical College Hospital, Dhaka, Bangladesh; ^3^Infectious Diseases Division, icddr,b, Dhaka, Bangladesh

## Abstract

*Chromobacterium violaceum* is a Gram-negative bacterium, found in tropical and subtropical regions. *C*. *violaceum* infection rarely occurs, but once occurs, it is associated with significant mortality due to severe systemic infection. Since the first human case from Malaysia in 1927, >150 cases of *C*. *violaceum* infection have been reported worldwide. We have described here a fatal case of *C*. *violaceum* infection in a tertiary care hospital in Dhaka, Bangladesh. To the best of our knowledge, this is the first case of *C*. *violaceum* infection in Bangladesh.

## 1. Introduction

*C*. *violaceum* is a Gram-negative, facultatively anaerobic, motile, nonsporing, capsulated coccobacillus. It usually produces an antioxidant pigment called violacein that gives the bacterial colonies purple color on agar medium. *C*. *violaceum* was first identified in 1881, and its pathogenic potential was first described by Wooley in 1905, when it was isolated from a fatal infection in a buffalo [1]. The first case of human infection was reported from Malaysia in 1927 [2]. People get infected by *C*. *violaceum* through intake of contaminated water, exposure of wound and traumatic lesions to contaminated soil or water, urinary tract, or medical equipment in the hospital environment [3–6]. Although it is ubiquitous in soil and stagnant water of tropical and subtropical regions, human infections are very rare. However, sporadic cases of *C*. *violaceum* infection with a high fatality rate (>50%) have been reported from different geographical locations [6, 7]. *C*. *violaceum* may cause localized cutaneous lesion, urinary tract infection, pneumonia, severe sepsis with metastatic abscesses, and septic shock progressing rapidly to death due to multiorgan failure [4, 6]. *C*. *violaceum* is usually resistant to penicillin, narrow-spectrum cephalosporin, amoxicillin-clavulanic acid, and polymyxin B but sensitive to fluoroquinolones, carbapenems, cotrimoxazole, chloramphenicol, cefepime, aminoglycosides, amikacin, imipenem, piperacillin-tazobactam, gentamycin, tetracycline, and trimethoprim-sulfamethoxazole [6, 8–12]. We have reported here a case of *C*. *violaceum* infection in a 40-year-old female who had a fatal outcome. The bacterium was isolated from the swab taken from the patient end of the heat and moisture exchanger (HME) filter, which was placed in between the endotracheal tube and mechanical ventilator.

## 2. Case Presentation

**Figure 1:**
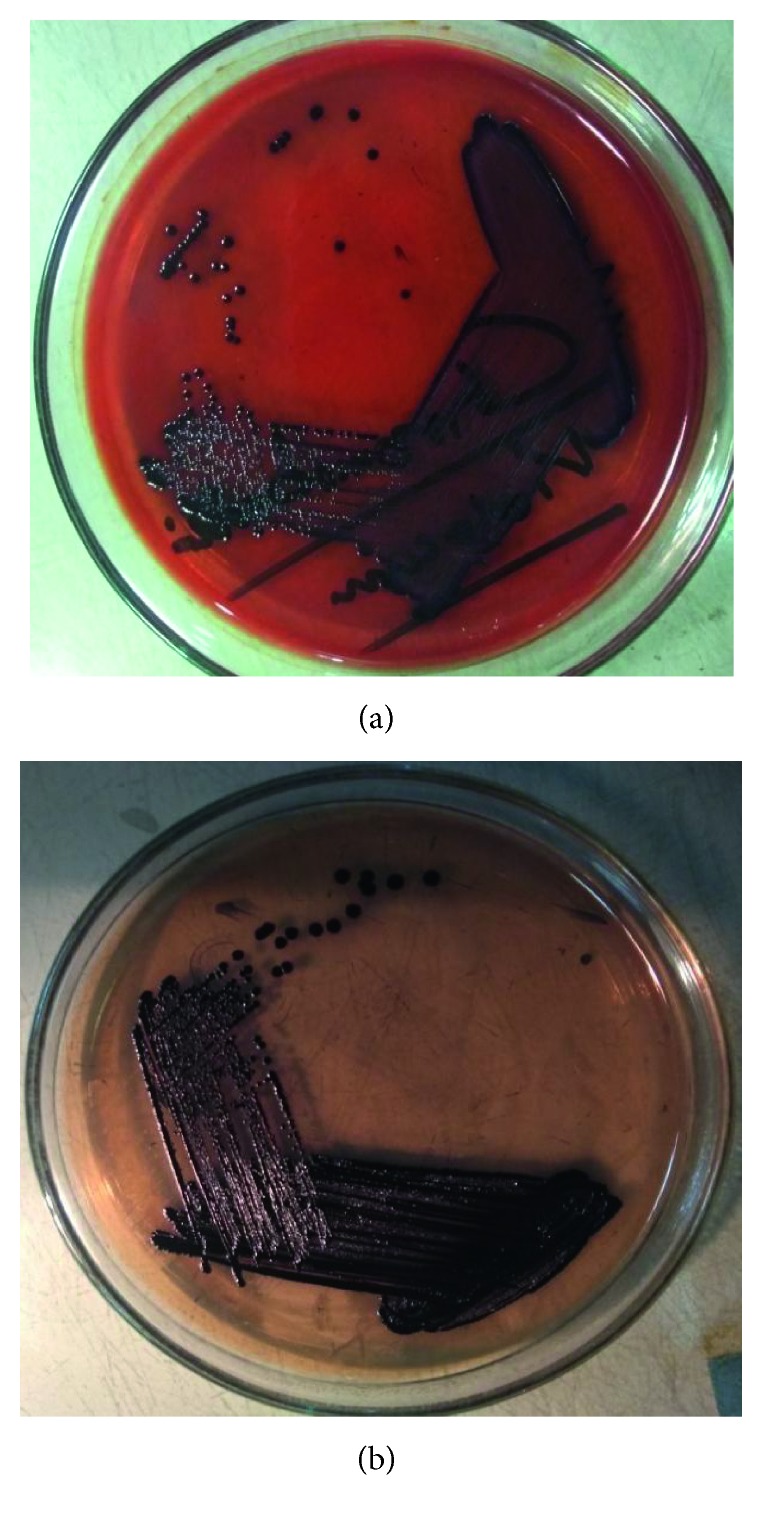
Violet-pigmented colonies on blood agar and MacConkey agar plates, typical of *C*. *violaceum*.

In June 2018, a woman aged 40 years was admitted to the Anwer Khan Modern Medical College Hospital (AKMMCH), Dhaka, with fever for 4 weeks, dry cough for 2 weeks, and yellow discoloration of urine and sclera for 3 days. She was given intravenous (IV) meropenem (1 g, 8 hourly), moxifloxacin (400 mg, OD), and steroid (hydrocortisone, 100 mg, 8 hourly) for 10 days by a primary caregiver before admission to AKMMCH. The patient was a homemaker and was residing in an urban area. She was hypertensive, but had no history of trauma, diabetes, or other immunodeficiency disorders. On admission, she had a blood pressure of 120/80 mmHg, pulse rate of 92 beats per minute, and respiratory rate of 22 breaths per minute. The fever with the highest recorded temperature of 103°F (39.4°C) was often associated with chills and rigor. Cough was associated with respiratory distress unrelated to exertion or posture. The initial diagnosis was pyrexia of unknown origin, and the patient was transferred to the intensive care unit (ICU). The patient was intubated and kept on mechanical ventilation as she developed pulmonary hemorrhage, hematuria, and septic shock (CRP: 98 mg/L, provided inotropic support). The patient was found to have pulmonary consolidation in chest X-ray, ascites and hepatoslpenomegaly in ultrasonogram, concentric left ventricular hypertrophy in echocardiogram, and type-2 respiratory failure in arterial blood gases. The patient was negative for HBsAg, anti-HBcAb, and anti-HCVAb. Hemoglobin level, lymphocyte count, and platelet count were much lower, while neutrophil count was higher than the reference ranges. Random blood sugar was in the normal level. However, other blood chemistry parameters, e.g., bilirubin, alanine transaminase (ALT), aspartate transaminase (AST), albumin, prothrombin time, C-reactive protein (CRP), lactate, procalcitonin, and lactate dehydrogenase (LDH) were remarkably higher (Table 1). Based on these observations, the final diagnosis was pneumonia with acute respiratory distress syndrome along with disseminated intravascular coagulation. The antimicrobial regimen of IV cefepime (1 g, BD) and meropenem (1 g, 8 hourly) was empirically initiated. Blood, urine, and two samples of swab from the HME filter, one from the patient end and one from the machine end, were inoculated on sheep blood agar and MacConkey agar plates. After 24 hours of aerobic incubation at 37°C, there was no growth in blood, urine, and swab from the machine end of the HME filter. However, small colonies with dark violet metallic pigmentation were observed on both blood agar and MacConkey agar plates (Figure 1) from the swab of the patient end of the HME filter. The violet pigmentation led us to assume that these were *C*. *violaceum* colonies. Identification of *C*. *violaceum* was confirmed by biochemical tests, e.g., triple sugar iron (TSI) agar reaction and catalase and oxidase tests [13]. The antibiotic susceptibility test of the isolates was performed for amikacin, gentamicin, ceftriaxone, ciprofloxacin, cotrimoxazole, piperacillin-tazobactam, and meropenem by Kirby–Bauer disk diffusion method on Mueller–Hinton agar, and the result was interpreted according to the Clinical and Laboratory Standards Institute (CLSI) guidelines for non-Enterobacteriaceae Gram-negative bacteria [14]. The antibiotic sensitivity report revealed that the isolate was susceptible to all antimicrobials, and the antimicrobial regimen was switched to IV ceftriaxone (1 g, 12 hourly), pipercillin (4.5 g, 6 hourly), and cotrimoxazole (960 mg, 12 hourly). However, there was no clinical improvement; the patient progressively went into multiorgan failure and died.

## 3. Discussion

Although human infections caused by *C*. *violaceum* are uncommon, more than 150 cases have been reported worldwide [4]. The majority of cases have been reported from the region of Americas, the East Western Pacific, and the Southeast Asia [6]. There have been few cases of *C*. *violaceum* infection in Nepal and India [8–12, 15–20]. However, to the best of our knowledge, this is the first case of *C*. *violaceum* infection reported from Bangladesh.

The most common mode of transmission of *C*. *violaceum* is the exposure of wounds and traumatic lesions to soil and stagnant or slow-flowing water containing the organism [4, 6]. The patient could not recall any history of trauma or wound. She also did not encounter unusual routes of exposure including scuba diving or near drowning or surgical procedures [21–23]. Medical device could be the source of infection, since it is a potential nosocomial pathogen [5, 24]. Patients with chronic granulomatous disease, severe glucose-6-phosphate dehydrogenase deficiency, and neutrophil dysfunction are generally susceptible to *C*. *violaceum* infection, indicating immunodeficiency as a predisposing factor [25, 26]. *C*. *violaceum*-induced sepsis has also been reported in patients with uncontrolled diabetes and systemic lupus erythematosus [8, 17]. The patient in the present case had no such history of immunodeficiency, and tests for HIV and TB were not performed. In fact, a review of 106 cases by Yang and Li demonstrated that the majority of the *C*. *violaceum*- infected patients were immunocompetent [6]. The clinical manifestations of *C*. *violaceum* infections are associated with localized skin lesion, gastrointestinal tract infection, urinary tract infection, pneumonia, multiple abscesses in the vital organs, hemophagocytic syndrome, respiratory distress syndrome, and fatal septicemia [6, 7]. In the present case, the patient developed pneumonia with acute respiratory distress syndrome due to pulmonary hemorrhage and disseminated intravascular coagulation.

As the patient was on mechanical ventilation, swabs from the patient end and the machine end of the HME filter were cultured. The machine end of the HME culture yielded no growth, which ruled out the possibility of ventilator-associated pneumonia. Isolation of *C*. *violaceum* from the patient end of the HME filter rather indicated the presence of community-acquired pneumonia. No bacteria were isolated from blood culture. Blood culture may have been negative due to extensive treatment with empirical antibiotics. However, the persistent toxic shock might lead to multiorgan failure and resulting death.

In line with previous studies, the present case outcome reflects that a high clinical suspicion, rapid diagnosis, and initiation of early antimicrobial therapy are critical to effectively manage this emerging and life-threatening infection, especially in patients with signs of sepsis.

## Figures and Tables

**Table 1:** Hematology and biochemical test results.

Test parameters	Unit	Results	Reference value (female)
Hemoglobin	gm/dl	7.9	11.5–16.5
White blood cell count	Cells per mm^3^	9500	5,000–10,000
Neutrophil	%	91	40–75
Lymphocyte (%)	%	07	20–45
Platelet count	Cells per mm^3^	80,000	200,000–500,000
Random blood sugar	mmol/L	6.0	<7.8
Bilirubin	mg/dl	8.0	0.2–1.2
Alanine transaminase (ALT)	U/L	363	0.01–31
Aspartate transaminase (AST)	U/L	405	0.01–32
Albumin	gm/dl	25	3.4–5.4
Prothrombin time	Second	18.3	11–13.5
C-reactive protein (CRP)	mg/L	98	<3
Lactate	mmol/L	4.27	0.56–2.22
Procalcitonin	ng/ml	47	<0.05
Lactate dehydrogenase (LDH)	U/L	597	100–190
